# ChIP-Atlas 2021 update: a data-mining suite for exploring epigenomic landscapes by fully integrating ChIP-seq, ATAC-seq and Bisulfite-seq data

**DOI:** 10.1093/nar/gkac199

**Published:** 2022-03-24

**Authors:** Zhaonan Zou, Tazro Ohta, Fumihito Miura, Shinya Oki

**Affiliations:** Department of Drug Discovery Medicine, Kyoto University Graduate School of Medicine, 53 Shogoin Kawahara-cho, Sakyo-ku, Kyoto 606-8507, Japan; Kyoto University Graduate Program for Medical Innovation, Yoshida-Konoe-cho, Sakyo-ku, Kyoto 606-8501, Japan; Kyoto University Graduate Division, Yoshida-Nihonmatsu-cho, Sakyo-ku, Kyoto 606-8501, Japan; Database Center for Life Science, Joint Support-Center for Data Science Research, Research Organization of Information and Systems, Yata 1111, Mishima, Shizuoka 411-8540, Japan; Department of Biochemistry, Kyushu University Graduate School of Medical Sciences, 3-1-1 Maidashi, Higashi-ku, Fukuoka 812-8582, Japan; Department of Drug Discovery Medicine, Kyoto University Graduate School of Medicine, 53 Shogoin Kawahara-cho, Sakyo-ku, Kyoto 606-8507, Japan; Precursory Research for Embryonic Science and Technology, Japan Science and Technology Agency, 4-1-8 Honcho, Kawaguchi, Saitama 332-0012, Japan

## Abstract

ChIP-Atlas (https://chip-atlas.org) is a web service providing both GUI- and API-based data-mining tools to reveal the architecture of the transcription regulatory landscape. ChIP-Atlas is powered by comprehensively integrating all data sets from high-throughput ChIP-seq and DNase-seq, a method for profiling chromatin regions accessible to DNase. In this update, we further collected all the ATAC-seq and whole-genome bisulfite-seq data for six model organisms (human, mouse, rat, fruit fly, nematode, and budding yeast) with the latest genome assemblies. These together with ChIP-seq data can be visualized with the Peak Browser tool and a genome browser to explore the epigenomic landscape of a query genomic locus, such as its chromatin accessibility, DNA methylation status, and protein–genome interactions. This epigenomic landscape can also be characterized for multiple genes and genomic loci by querying with the Enrichment Analysis tool, which, for example, revealed that inflammatory bowel disease-associated SNPs are the most significantly hypo-methylated in neutrophils. Therefore, ChIP-Atlas provides a panoramic view of the whole epigenomic landscape. All datasets are free to download via either a simple button on the web page or an API.

## Graphical Abstract

**Graphical Abstract:**
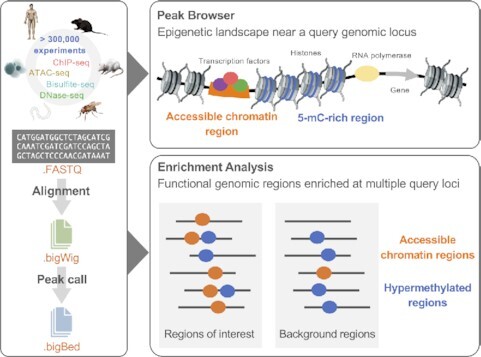
ChIP-Atlas 2021 update integrates all publicly available ATAC-seq and WGBS data besides ChIP-seq data for six representative model organisms, facilitating comprehensive exploration of chromatin accessibility, DNA methylation status, and protein binding around single or multiple genes and genomic regions of users' interest.

## INTRODUCTION

In the past decade, despite the increasing number of high-throughput sequencing experiments, the secondary use of the obtained raw data has required complex and large-scale computational processing, and thus most data are still being hoarded. Since 2015, we have been comprehensively collecting, analyzing and integrating almost all chromatin immunoprecipitation sequencing (ChIP-seq) (1) and DNase-seq (2)—a method for profiling chromatin regions accessible to DNase—data derived from six representative model organisms (*Homo sapiens*, *Mus musculus*, *Rattus norvegicus*, *Drosophila melanogaster*, *Caenorhabditis elegans* and *Saccharomyces cerevisiae*) archived at the Sequence Read Archive (SRA). SRA is the largest publicly available data repository that accepts submissions of high-throughput sequencing data, which is maintained by NCBI, EBI, and DDBJ. Data-mining tools powered by these data were provided through our web server, ChIP-Atlas (https://chip-atlas.org) (3), for visualization of assembled peak data and enrichment analysis for given genomic loci to identify transcription factor (TF) binding and histone modification status. ChIP-Atlas is powered by dedicated manual curation and annotation of experimental metadata, and a uniform data process pipeline to reveal the complex architecture of the transcription regulatory landscape.

Information on TF binding and histone marks is, however, still insufficient to fully understand the regulatory systems for gene expression control because an absence of nucleosomes and low methylation levels also characterize active promoters, enhancers, and other gene regulatory sequences (4–7). To detect accessible chromatin regions, several experimental methods including DNase-seq, formaldehyde-assisted isolation of regulatory elements (8), and assay for transposase-accessible chromatin with sequencing (ATAC-seq) (9) have been developed. Among these, ATAC-seq identifies accessible chromatin regions based on their increased accessibility to Tn5 transposase integration and is now predominantly used given its advantages of technical ease and sensitivity to a small number of cells. In contrast, bisulfite sequencing (Bisulfite-seq) is a well-established protocol to detect methylated cytosines in genomic DNA, which employs a chemical method that selectively deaminates unmodified cytosine to uracil while leaving 5-methylcytosine intact before DNA sequencing (10).

Here, we describe the ChIP-Atlas 2021 update, which collected all the ATAC-seq (*n* = 66 104) and whole-genome bisulfite sequencing (WGBS) data (*n* = 51 074) for the six representative model organisms. This update makes ChIP-Atlas, to our knowledge, the first and only web service that enables users to explore not only protein binding, but also chromatin accessibility and DNA methylation status, within a single given genomic region of interest (ROI) at the same time (Peak Browser tool). ChIP-Atlas can also be used to reveal the regulatory network involved in a batch of genomic ROIs (Enrichment Analysis tool) based on ATAC-seq and WGBS data in addition to ChIP-seq data in the previous version. ChIP-Atlas provides a panoramic view of the whole epigenomic landscape and should be of great interest to researchers in the fields of genetics and genomics, as well as those studying transcriptional regulation in general.

## MATERIALS AND METHODS

### Source data obtained from SRA

We obtained sample metadata from the NCBI BioSample database FTP server (ftp://ftp.ncbi.nlm.nih.gov/biosample). We also retrieved the metadata of SRA experiments (accession number with the prefix SRX, ERX or DRX; hereafter SRXs), such as library preparation or used sequencing instrument models, from the NCBI SRA FTP server (ftp://ftp.ncbi.nlm.nih.gov/sra/reports/Metadata). ChIP-Atlas uses ATAC-seq and WGBS SRXs that meet the following criteria: LIBRARY_STRATEGY is ‘ATAC-seq’ or ‘Bisulfite-seq’; LIBRARY_SOURCE is ‘GENOMIC’; LIBRARY_SELECTION is ‘PCR’ or ‘others’ for ATAC-seq, and ‘RANDOM’ for WGBS; taxonomy_name is ‘*Homo sapiens*,’ ‘*Mus musculus*’, ‘*Rattus norvegicus*’, ‘*Caenorhabditis elegans*’, ‘*Drosophila melanogaster*’ or ‘*Saccharomyces cerevisiae*’; and INSTRUMENT_MODEL includes ‘Illumina’, ‘NextSeq’ or ‘HiSeq.’ For ChIP-seq and DNase-seq data, refer to the previous paper on ChIP-Atlas (3).

### Primary processing

Binarized sequence raw data (.sra) for each SRX were downloaded and decompressed into FASTQ format with the ‘fasterq-dump’ command of SRA Toolkit (ver. 2.9.4; https://ftp-trace.ncbi.nlm.nih.gov/sra/sdk/2.9.4/sratoolkit.2.9.4-ubuntu64.tar.gz) according to the default mode, with the exception of paired-end reads, which were decoded with the ‘-split-files’ option. For ATAC-seq data, the subsequent alignment (with Bowtie2 [ver. 2.2.2] (11)) and peak call (with MACS2 [ver. 2.1.1; macs2 callpeak] (12)) process was the same as that for ChIP-seq and DNase-seq data, which was previously described in the first ChIP-Atlas paper. As for WGBS data, BMap (ver. 1.0; https://github.com/FumihitoMiura/Project-2/blob/master/Project-2.tar.gz) (13), a speedy aligner with small temporary file sizes, was used for alignment and MethPipe (ver. 4.1.1; https://github.com/smithlabcode/methpipe/releases/download/v4.1.1/methpipe-4.1.1.tar.gz) (14) was subsequently used as a region-caller for identifying hyper-, partially and hypo-methylated regions (MRs). After creation of the index files for all genome assemblies, we aligned the downloaded FASTQ files against the reference genomes with the ‘-fastq’ and ‘-pfastq’ options for single-end and paired-end reads, respectively. The alignment data (in bigWig format) containing methylation level and coverage information were generated for each SRX. Methylation level and coverage were then calculated for each CpG, and these data were next streamed into MethPipe. The ‘hypermr,’ ‘pmd,’ and ‘hmr’ sub-commands of MethPipe were used for identifying hyper-, partially, and hypo-MRs (in bigBed format), respectively, for each SRX according to the default mode.

### Analysis of disease-associated SNPs

GWAS SNP data were downloaded from the website of UCSC genome browser (https://hgdownload.soe.ucsc.edu/goldenPath/hg19/database/gwasCatalog.txt.gz; on 21 December 2021) (15). A BED format file was created by extending the genomic coordinates of all SNPs by 5 kb up- and downstream. Genomic regions containing SNPs were then categorized into ‘inflammatory bowel disease (IBD)-associated’ (*n* = 393) and ‘Others’ (*n* = 189 036) according to the disease/phenotype names listed in the ‘trait’ column. The IBD-associated SNPs (in BED format) were directly used as ‘dataset A’ for the Enrichment Analysis tool on the ChIP-Atlas web interface. Meanwhile, 3930 SNPs in the ‘Others’ category, which is ten times the number of IBD-associated SNPs, were randomly selected as the background data for ‘dataset B’.

## RESULTS

### Overview of the ChIP-Atlas update

**Figure 1.**
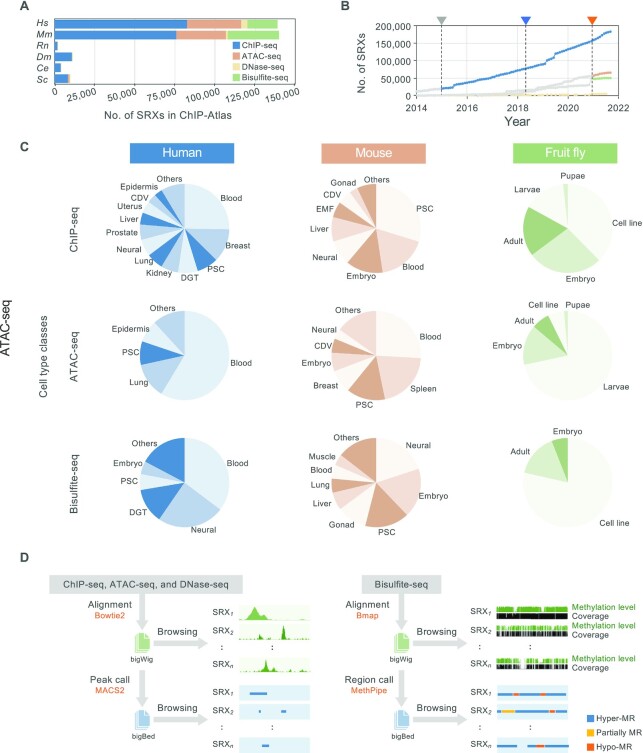
Overview of the ChIP-Atlas data set and computational processing. (**A**) Numbers of ChIP-seq, DNase-seq, ATAC-seq and WGBS experiments recorded in ChIP-Atlas (as of September 2021). Colors indicate different experiment types. Color legend is shown in the figure. *Hm, Homo sapiens; Mm, Mus musculus; Rn, Rattus norvegicus; Dm, Drosophila melanogaster; Ce, Caenorhabditis elegans; Sc, Saccharomyces cerevisiae*. (**B**) Cumulative number of SRX-based experiments recorded in ChIP-Atlas. Colors of the dots indicate different experiment types. Color legend is the same as that in (A). The gray, blue, and orange arrowheads indicate the timing of the release of ChIP-Atlas in 2015, the publication of the first ChIP-Atlas paper in 2018, and the inclusion of ATAC-seq and WGBS in 2021, respectively. ChIP-seq and DNase-seq data published before 2015, and ATAC-seq or WGBS data published before January 2021 are shown in gray. (**C**) Numbers of experiments according to cell type classes for human, mouse, and fruit fly data. PSC, pluripotent stem cell; CDV, cardiovascular; DGT, digestive tract; EMF, embryonic fibroblast. (**D**) Overview of data processing. Raw sequence data are downloaded from NCBI SRA, aligned to a reference genome, and subjected to peak calling, all of which can be monitored with the IGV genome browser. MR, methylated region.

In this update, the major expansion of the experiment types results in significant growth in the number of experiments and the number of annotated functional genome regions by including all ATAC-seq and WGBS datasets archived at SRA (Figure 1A, Table 1). For unified management of the records, each experiment is assigned a unique ID (hereafter referred to as ‘SRX’) in ChIP-Atlas, which is exactly the same as the original SRA accession number. The number of SRXs collected in ChIP-Atlas is over 300 000 for the six organisms, which corresponds to 84.3% of the total number of ChIP-seq, ATAC-seq, DNase-seq and Bisulfite-seq SRXs in SRA for all of these organisms (*n* = 362 121 as of September 2021; Table 1). In this update, we adopted the latest version of genome assemblies in addition to previous ones (Table 2). Since the public release of ChIP-Atlas, the data have been updated monthly concurrent with the monthly update of the NCBI SRA metadata dump (Figure 1B), by which >100 000 ChIP-seq experimental datasets have been added since 2018 (Table 1).

**Table 1.** Data statistics of ChIP-Atlas

Experiment type	ChIP-seq		ATAC-seq	DNase-seq	Bisulfite-seq	All
Year	2021	2018	2021	2021	2021	2021
Number of experiments	182 891	74 076	66 104	5346	51 074	305 415
Number of ChIP antigens	2 477	1 979	NA	NA	NA	2 477
Number of cell types	2 486	1 615	772	406	553	2 918
Number of peaks	1 329 049 688	717 206 934	362 032 813	128 597 931	3 798 954 139	5 618 634 571

**Table 2.** Comparison of ChIP-Atlas with other similar web services

	ChIP-Atlas	Cistrom DB	ReMap	GTRD	MethBank
Data source	NCBI SRA	GEO, ENCODE, and Roadmap Epigenetics	GEO, ENCODE, and ENA	GEO, SRA, ENCODE, and modENCODE	NCBI SRA and Genome Sequence Archive
Experiments	ChIP-seq, **ATAC-seq**, DNase-seq, and **Bisulfite-seq**	ChIP-seq, ATAC-seq, and DNase-seq	ChIP-seq, ChIP-exo, and DAP-seq	ChIP-seq, ChIP-exo, ChIP-nexus, MNase-seq, DNase-seq, FAIRE-seq, ATAC-seq, and RNA-seq	Bisulfite-seq
Filtering of data for quality control	No	Yes	Yes	No	Yes
Number of experiments	76 217 → **305 415**	56 442	19 983	36 540	673
Organism	Hs, Mm, Rn, Dm, Ce, and Sc	Hs and Mm	Hs, Mm, Dm and At	Hs, Mm, At, Ce, Dr, Dm, Rn, Sc and Sp	Hs, Dr, Mm, Os, Gm, Me, Pv and Sl
Genome assembly	hg19, **hg38**, mm9, **mm10**, rn6, dm3, **dm6**, ce10, **ce11** and sacCer3	hg38 and mm10	hg38, mm10, dm6, and tair10 (can be lifted to hg19/mm9 with liftover)	hg38, mm10, tair10, ce11, danRer11, dm6, rn6, sacCer3 and spo2	hg38, danRer7, mm10, IRGSP-1.0, Gmax_275_v2.0, Mesculenta_305_v6, Pvulgaris_218_v1 and GCF_000188115.3_SL2.53
Alignment tool	Bowtie2/**BMap**	BWA	Bowtie2	Bowtie2	WSBA
Peak caller	MACS2/**MethPipe**	MACS2	MACS2	MACS2, MACS, GEM, PICS, SISSRs	NA
Display format for each experiment	Alignment and peaks	Alignment and peaks	Peaks	Peaks	Alignment
Browsing assembled peaks	Possible	None	Possible	Possible	NA
Genome browser	IGV and UCSC	WashU and UCSC	UCSC, ENSEMBL and IGV	Self-developed, UCSC and ENSEMBL	JBrowse
Integrative analysis tools	Search tool for target genes and colocalizing factors of given TF, and enrichment analysis tool for given genes and genomic coordinates	Search tool for target genes of single experiment, TFs binding to single given genomic locus or query gene	Enrichment analysis tool for given genomic coordinates relative to random background	Search tool for target genes of given TF	Predictor of DNA methylation, age of human blood and enrichment analysis tool for identification of differentially methylated promoters

*Hm*, *Homo sapiens*; *Mm*, *Mus musculus*; *Rn*, *Rattus norvegicus*; *Dm*, *Drosophila melanogaster*; *Ce*, *Caenorhabditis elegans*; *Sc*, *Saccharomyces cerevisiae*; *At*, *Arabidopsis thaliana*; *Dr*, *Danio rerio*; *Sp*, *Schizosaccharomyces pombe*; *Os*, *Oryza sativa*; *Gm*, *Glycine max*; *Me*, *Manihot esculenta*; *Pv*, *Phaseolus vulgaris*; *Sl*, *Solanum lycopersicum*.

We manually curated and annotated the cell types used in each experiment according to commonly or officially adopted nomenclature. The cell types were further categorized into superordinate ‘cell type classes’ (Figure 1C). We adapted a uniformed data process pipeline (Figure 1D; detailed in the Materials and Methods), in which the ChIP-seq, ATAC-seq, and DNase-seq data are aligned to corresponding reference genomes with Bowtie2 (11) and then subjected to peak calling with MACS2 (12); meanwhile, for WGBS data, we align the raw reads against the reference genomes using BMap before applying MethPipe for statistically calling hyper-, partially, and hypo-methylated regions (MRs). All alignment and peak-call data are freely downloadable from http://dbarchive.biosciencedbc.jp/kyushu-u/ (detailed on the documentation page of ChIP-Atlas [https://github.com/inutano/chip-atlas/wiki/#downloads_doc]), and can be browsed by users in the IGV genome browser (16) by entering the SRX ID or a given keyword (or keywords) in the corresponding Data Search page of ChIP-Atlas (Supplementary Figure S1 and Supplementary Material S1).

### Example of use

#### Peak browser

**Figure 2.**
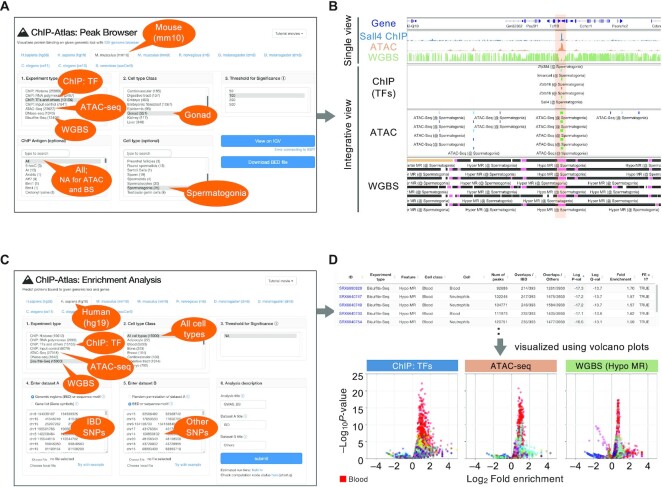
Examples of use of the integrative analysis tools in ChIP-Atlas. (**A**) Parameters when users perform a query to show chromatin accessibility, methylation status, and TF binding around mouse *Tcf19–Cchcr1* locus in spermatogonia using the Peak Browser tool. (**B**) Peak-call data for TF ChIP-seq, ATAC-seq and WGBS SRXs around the mouse *Tcf19–Cchcr1* locus are shown in the IGV genome browser. The highlighted region indicates an accessible chromatin and hypo-methylated region. Bars represent the peak regions, with the curated names of the ChIP antigens (only for ChIP-seq peaks) and cell types being shown below the bars. The color of the bars in the ‘ChIP (TFs)’ and ‘ATAC’ panel indicates the score calculated with the peak-caller MACS2 (−log_10_[*Q*-value]). Black, pink, and beige bars in the ‘WGBS’ panel indicate hyper-, hypo- and partially methylated regions, respectively. (**C**) Parameters when users perform a query to evaluate TF binding, chromatin accessibility, and methylation levels of multiple IBD-associated SNPs in all cell types. (**D**) The resultant HTML of enrichment analysis using WGBS data (upper). Volcano plots for visualizing the results (bottom). X-axis, log_2_[fold enrichment]; Y-axis, –log_10_[*P*-value]. Each dot represents an individual SRX. SRXs are categorized by cell type classes and indicated by different colors. Red, ‘Blood’ class. The whole color legend is summarized in Supplementary Table S2.

All peak-call data recorded in ChIP-Atlas can be presented graphically with the Peak Browser tool. One can therefore easily understand not only protein–genome interactions (ChIP-seq), but also chromatin accessibility (ATAC-seq) and DNA methylation levels (WGBS), within any query genomic region of interest (ROI). To implement this tool, we integrated a large amount of peak-call data, indexed them for IGV, and constructed a web interface that externally controls IGV preinstalled on the user's machine (tested on Mac, Windows and Linux platforms). For instance, upon the specification of ChIP-seq, ATAC-seq or WGBS data of mouse spermatogonia on the web page (Figure 2A), the corresponding results are automatically streamed into IGV (Figure 2B). In this case, multiple ATAC-seq and WGBS in spermatogonia data characterize an accessible chromatin region and hypo-MR, respectively, at the locus between the *Tcf19* and *Cchcr1* genes, where multiple factors such as Smarca4, Zbtb16, and Sall4 are colocalized, suggesting that the *Tcf19*–*Cchcr1* locus is robustly hypo-methylated and open to bind Sall4 and other TFs in spermatogonia. Representative ChIP-seq (SRX1284250), ATAC-seq (SRX5884282) and WGBS (SRX749893) alignment data are also exhibited in the top panel of Figure 2B. The IGV sessions can be saved as XML files at any moment, so that the results obtained by using the Peak Browser tool can be easily and precisely shared among collaborators (Supplementary Material S2). With the use of ChIP-Atlas, users can not only check individual experimental data, but also browse an integrative landscape of multiple epigenetic profiling results, potentially providing useful insight into the location of functional genomic regions (enhancers, promoters and insulators) and the corresponding regulators.

#### Enrichment analysis

Enrichment Analysis is a tool that allows a search for TFs, accessible chromatin regions, and hypo- or hyper-MRs enriched at a batch of genomic ROIs or gene loci. Upon the submission of two sets of genomic regions (ROIs and background regions), all SRXs are evaluated to count the overlaps between the peaks and submitted regions and to perform Fisher's exact test, before returning the enrichment analysis results in HTML and TSV formats. Although ChIP-Atlas can generate random background regions for comparison to ROIs, the users are strongly recommended to provide biologically appropriate background genomic intervals because most randomly selected regions are probabilistically devoid of any functional genomic annotation. The results are assigned unique URLs, which are permanently available to the public. As an example of usage, we selected inflammatory bowel disease (IBD)-associated SNPs identified by GWAS as the ROIs (*n* = 393) and other SNPs as the background (*n* = 3930), and we applied these selections to the Enrichment Analysis tool (Figure 2C; detailed in the Materials and Methods). The results in HTML format are shown in Figure 2D (upper), including SRX IDs (column 1), features (column 3), cell types (column 5), *P*-values (column 9) and fold enrichments (column 11), where hypo-MRs of neutrophils are significantly enriched (*P* = 1 × 10^−17^). To visually interpret the results, we downloaded the resultant TSV files (refer to Supplementary Table S1 for unique URLs for each analysis) and generated volcano plots (Figure 2D [bottom]; Supplementary Table S1). The IBD-associated SNPs were shown to be enriched in hypo-MRs and accessible chromatin regions of blood-related cells (red in Figure 2D), including the ATAC-seq peaks of macrophages (*P* = 1 × 10^−20^) and Th17 cells (*P* = 1 × 10^−17^), both involved in the inflammation of IBD. Furthermore, the enrichment analysis of TF ChIP-seq data revealed that the IBD-associated SNPs were preferentially bound by STAT1 in monocytes (*P* = 1 × 10^−22^) and SPI1 in macrophages (*P* = 1 × 10^−21^), essential factors for inflammation and macrophage differentiation, respectively, which is consistent with the nature of IBD as an autoimmune disease (17). All resultant URLs for generating Figure 2D are summarized in Supplementary Table S2. API of the Enrichment Analysis tool is also provided by ChIP-Atlas, the general instructions for which can be found on the documentation page (https://github.com/inutano/chip-atlas/wiki/Perform-Enrichment-Analysis-programmatically).

## DISCUSSION

In this paper, we present a major update of ChIP-Atlas involving significant expansion in the number of experiments by including all public ATAC-seq and WGBS data. The updated web service enables users to explore not only protein binding, but also chromatin accessibility and DNA methylation levels within single (Peak Browser tool) or multiple (Enrichment Analysis tool) queries of genomic ROIs or gene loci. As an example of use, we performed enrichment analysis to reveal that IBD-associated SNPs are the most significantly hypo-methylated in the neutrophils, a granulocyte subtype known to be involved in autoinflammatory IBD (17).

Before and after the public release of ChIP-Atlas, several similar web services have been released (Table 2). For ChIP-seq, ATAC-seq and DNase-seq data, Cistrome DB (http://cistrome.org/db/), ReMap (https://remap2022.univ-amu.fr/), and GTRD (https://gtrd.biouml.org/) are representatives providing thousands of preprocessed datasets (18–20). As for Bisulfite-seq data, MethBank (http://bigd.big.ac.cn/methbank) is widely used (21). ChIP-Atlas covers much more experimental data than all of these other services. ChIP-Atlas does not cover ChIP-exo (22) and MNase-seq (23) data, in contrast to ReMap and GTRD, and there are fewer available organisms than in GTRD and MethBank. Alignment data (in bigWig format) are available from ChIP-Atlas, Cistrome DB and MethBank. Integrative analysis tools are provided in all services. Enrichment analysis is possible with ChIP-Atlas in both a GUI and an API, while ReMap provides such a tool in a CLI (R package named ‘ReMapEnrich’). Since the publication of the first ChIP-Atlas paper, we have improved the service to make it compatible with the latest versions of reference genomes such as hg38 for human and mm10 for mouse, as is the case for other services. Meanwhile, alignment of raw reads against old references is still performed during the monthly update of ChIP-Atlas, and alignment and peak-call data in old versions are still provided for analyzing the data of users obtained years ago.

In addition to the examples of use mentioned in the RESULTS section, ChIP-Atlas has been cited by hundreds of peer-reviewed articles since its first release in 2015, including research for analyzing cis-regulatory elements of certain genes (24,25), and TF enrichment at genomic ROIs and query genes (26–30) (see http://chip-atlas.org/publications for the full list of publications citing ChIP-Atlas). Furthermore, because all alignment (bigWig) and peak-call (bigBed) data can be freely downloaded, ChIP-Atlas is now interconnecting with many other databases or web services such as UCSC Browser, DeepBlue (an epigenomic data server providing a central data access hub for large collections of epigenomic data), RegulatorTrail (a web service predicting target genes of TFs), jPOSTrepo (a data repository of sharing raw/processed mass spectrometry data), and the Signaling Pathways Project (a multi-omics knowledgemine based upon public transcriptomic and cistromic datasets) (31–35). Along with the inclusion of ATAC-seq and WGBS data, and the ongoing monthly updates with semiautomatic pipelines and systematic curation, the source data in ChIP-Atlas are continuously expanding. We are planning to include more experiment types such as CUT&Tag (36) and ChIL-seq (37) and more organisms including plants such as *Arabidopsis thaliana*. Integration of preprocessed 3D genome conformation data such as Hi-C datasets (38) into the Peak Browser and Enrichment Analysis tool is also on the agenda.

## DATA AVAILABILITY

ChIP-Atlas (https://chip-atlas.org) is a publicly available web server with no sign up required. Documentation for data processing and downloadable data are available in the ‘Documentation’ section (https://github.com/inutano/chip-atlas/wiki).

## Supplementary Material

gkac199_Supplemental_Files
