# Supplementary material for: ChIP-Atlas 2021 update: a data-mining suite for exploring epigenomic landscapes by fully integrating ChIP-seq, ATAC-seq and Bisulfite-seq data
Source: Nucleic Acids Res. 2022 Mar 24;50(W1):W175–82. doi: 10.1093/nar/gkac199 (PMC9252733; doi:10.1093/nar/gkac199)
Supplement: gkac199_Supplemental_Files [file gkac199_supplemental_files.zip › Supplementary_Legend.docx]

**SUPPLEMENTARY TABLE AND** **FIGURE LEGENDS**

Supplementary Table S1. URLs for the results of enrichment analysis described in Figure 2C and D. Links of results in HTML and TSV formats are listed.

Supplementary Table S2. Color legend for Figure 2D. Column 1, Cell type class; column 2, Color name; column 3, HTML color code.

Supplementary Figure S1. Method for downloading alignment and peak-call data from the ChIP-Atlas website. **(A)** Screenshot of Data Search page when searching data by given keywords. **(B)** Summary page for a single WGBS SRX. Methylation rate, coverage, and hyper-, partially, and hypo-methylated region data can be downloaded by simply clicking corresponding buttons. **(C)** Downloaded bigWig and bigBed files specified in (B) were visualized with IGV.
